# Inequities in dietary intake and eating behaviours among adolescents in Canada

**DOI:** 10.17269/s41997-024-00854-0

**Published:** 2024-02-21

**Authors:** Karen A. Patte, Markus J. Duncan, Angelica Amores, Emily Belita, Rita Kocsis, Negin A. Riazi, Rachel Laxer, Scott T. Leatherdale

**Affiliations:** 1 Department of Health Sciences, Brock University, St. Catharines, ON Canada; 2 Children’s Hospital of Eastern Ontario Research Institute, Ottawa, ON Canada; 3 School of Public Health Sciences, University of Waterloo, Waterloo, ON Canada; 4 School of Nursing, McMaster University, Hamilton, ON Canada; 5 Student Health & Wellbeing, University of British Columbia, Vancouver, BC Canada; 6 Public Health Ontario, Toronto, ON Canada

**Keywords:** Healthy eating, Equity, Youth, Nutrition, Diet, COVID-19, Adolescent, Régime alimentaire sain, équité, jeunes, nutrition, régime, COVID-19, adolescent

## Abstract

**Objective:**

To provide contemporary evidence of how dietary intake and eating behaviours vary by social positions among adolescents.

**Methods:**

We used survey data collected during the 2020–2021 school year from 52,138 students attending 133 secondary schools in Alberta, British Columbia, Ontario, and Quebec, Canada. Multiple regression models tested whether self-reported indicators of dietary intake and eating behaviours differed by gender, race/ethnicity, and socioeconomic status (SES).

**Results:**

Females were more likely than males to skip breakfast, restrict eating, and consume fruit, vegetables, and fast food on more days. Gender-diverse/“prefer not to say” students were more likely to restrict eating than males and the least likely to consume breakfast and drink water daily, and fruits and vegetables regularly. Black and Latin American students were more likely to restrict eating and consume purchased snacks and fast food, and less likely to drink water daily than white and Asian adolescents. Daily breakfast consumption was most likely among Latin American students. Black students were the least likely to report eating breakfast daily and fruits and vegetables regularly. Lower SES was associated with lower odds of eating breakfast and drinking water daily and regular fruit and vegetable consumption, and higher odds of restrictive eating and purchased snack consumption. Fast food consumption had a u-shaped association with SES.

**Conclusion:**

Results emphasize gender, racial/ethnic, and socioeconomic inequities in the diets and eating behaviours of adolescents. There is a critical need to address the structural factors contributing to inequities and prevent the consequences of dietary disparities.

## Résumé

**Objectif:**

Montrer à l’aide de preuves contemporaines que les apports et les comportements alimentaires des adolescentes et des adolescents varient selon leur position sociale.

**Méthode:**

Nous avons utilisé des données d’enquête recueillies durant l’année scolaire 2020–2021 auprès de 52 138 élèves fréquentant 133 écoles secondaires en Alberta, en Colombie-Britannique, en Ontario et au Québec (Canada). Des modèles de régression multiple ont déterminé si des indicateurs autodéclarés d’apports alimentaires et de comportements alimentaires différaient selon le genre, la race/l’ethnicité et le statut socioéconomique (SSE).

**Résultats:**

Les filles étaient plus susceptibles que les garçons de sauter le petit déjeuner, de restreindre leur alimentation et de consommer des fruits, des légumes et des aliments de restauration rapide sur un plus grand nombre de jours. Les élèves de diverses identités de genre/« préférant ne pas répondre » à la question du genre étaient plus susceptibles de restreindre leur alimentation que les garçons; c’était aussi la population d’élèves la moins susceptible de prendre un petit déjeuner et de boire de l’eau tous les jours, et de consommer des fruits et légumes régulièrement. La population étudiante noire et latino-américaine était plus susceptible de restreindre son alimentation et de consommer des collations achetées et des aliments de restauration rapide, et moins susceptible de boire de l’eau tous les jours, que les adolescentes et les adolescents blancs et asiatiques. La consommation quotidienne du petit déjeuner était la plus probable chez les élèves latino-américains. Les élèves noirs étaient les moins susceptibles de déclarer prendre un petit déjeuner tous les jours et consommer des fruits et légumes régulièrement. Le SSE plus faible était associé à des probabilités moins élevées de prendre un petit déjeuner et de boire de l’eau tous les jours et de consommer des fruits et légumes régulièrement, et à des probabilités plus élevées de restreindre son alimentation et de consommer des collations achetées. La consommation d’aliments de restauration rapide présentait une association en U avec le SSE.

**Conclusion:**

Les résultats montrent qu’il existe des iniquités sur les plans du genre, de la race/l’ethnicité et du statut socioéconomique dans les régimes et les comportements alimentaires des adolescentes et des adolescents. Il existe un besoin pressant d’aborder les facteurs structurels qui contribuent à ces iniquités et de prévenir les conséquences des disparités alimentaires.

## Introduction

The accessibility of nutritious food varies within Canada and globally (United Nations, 2019). Recent global and national events, particularly the coronavirus disease 2019 (COVID-19) pandemic, reinvigorated calls to address the social stratification of health. Contemporary evidence of dietary intake and eating behaviours among young Canadians is needed to inform policy and practice for a more equitable future.

The release of the 2019 Canada’s Food Guide was met with concern about the ability of many Canadian families to follow the recommendations (Pannu & Hamann, 2019). The revised guidelines recommend regularly consuming fruits and vegetables, avoiding restrictive fad diets, swapping sugar-sweetened beverages with water, and replacing highly processed and prepared foods (e.g. fast food, snacks from convenience stores and vending machines) with meals planned and cooked at home (Health Canada, 2019). The diets of many young Canadians fall short; adolescents had the poorest dietary intake of all age groups in past national surveys, characterized by lower fruit, vegetable, and fibre intake; excess sodium and sugar; and a higher proportion of low nutrient energy-dense foods (Garriguet, 2009; Tugault-Lafleur & Black, 2019).

Promoting healthy diets and eating behaviours during adolescence is important to support growth and development, prevent chronic disease, and establish positive habits that often persist into adulthood. Sociocultural influences take greater importance during this developmental period, as adolescents engage in identity exploration and place increased priority on peer acceptance and comparisons. Young people are more exposed to food marketing, and weight-normative and diet culture messages (e.g. via social media), which vary by gender and race (Harris et al., 2021; Lucibello et al., 2021). However, while adolescents have increasing autonomy, their behaviours are still shaped by their family and home environment via factors such as socioeconomic status (SES) and culture (Neumark-Sztainer et al., 1999, 2003). Schools also play a role, with about one third of weekday caloric intake occurring during school hours (Tugault-Lafleur et al., 2017).

Many determinants of adolescents’ diets and eating behaviours were likely impacted by recent events, particularly the COVID-19 pandemic and enacted public health measures (e.g. lockdowns, physical distancing, school closures). Widespread concerns about both overnutrition and undernutrition, as well as disordered eating, arose in response (Pryor & Dietz, 2022; Paslakis et al., 2021; Zemrani et al., 2021). While some families may have had more time for regular home-cooked meals and less opportunities to purchase convenience foods, factors such as increased food prices, disrupted school nutrition programs, reduced structure/routine, and greater exposure to social media may have adversely impacted adolescent diets and eating behaviours. Indeed, both positive and negative dietary shifts have been reported. Systematic reviews found evidence of higher consumption of breakfast, snack food (e.g. chips), and sugary drinks among children and adolescents than before the lockdown (Brakspear et al., 2022; Mignogna et al., 2022; Viner et al., 2022). However, most research was during the early pandemic lockdown, no studies were from North America, few used adolescent samples, and outcomes primarily included dietary intake as opposed to eating behaviours (Brakspear et al., 2022; Mignogna et al., 2022; Viner et al., 2022). Recent Canadian research has primarily focused on parents and younger children, with reports of increased home cooking and snacking (Carroll et al., 2020; Jansen et al., 2021).

Experiences of the pandemic varied across and within population subgroups. Regions with greater proportions of racialized and lower SES families had elevated rates of COVID-19 and longer school closures, while more affluent and rural areas were less affected (Gallagher-Mackay et al., 2021). Economic impacts were also inequitably distributed, and contributed to increases in the prevalence and severity of food insecurity (Dondi et al., 2020). Existing research suggests dietary disparities increased over the pandemic; lower SES was associated with relatively less healthy shifts, including reduced fruit and vegetable intake and increased convenience food consumption and meal skipping (Brakspear et al., 2022; Mignogna et al., 2022; Viner et al., 2022). In a 2021 nationally representative survey of high school students in the United States, fruit, vegetable, and daily breakfast consumption decreased between 2019 and 2021, with disparities by gender, race, and ethnicity (Brakspear et al., 2022). Limited research exists among Canadian adolescents and beyond the early pandemic lockdown.

This study aimed to provide a contemporary profile of various indicators of dietary intake and eating behaviours by gender, race/ethnicity, and SES in a large sample of secondary school students in 2020–2021, the first full academic year post-COVID-19 onset.

## Methods

### Design and data collection

We used student-level data from the ongoing *Cannabis, Obesity, Mental health, Physical activity, Alcohol, Smoking, and Sedentary behaviour* (COMPASS) study (Leatherdale et al., 2014) collected during the 2020–2021 school year in Alberta, British Columbia, Ontario, and Quebec, Canada. COMPASS collects data annually from a rolling cohort of students attending a convenience sample of secondary schools. Given the ongoing COVID-19 pandemic response, COMPASS student questionnaires were administered online; schools emailed students a survey link and one reminder. COMPASS purposefully samples schools and school boards based on permitted use of active-information passive-consent parental permission protocols, given the importance for improving response and completion rates, particularly among more socioeconomically deprived populations (Spence et al., 2014). Using these protocols, schools communicated study information to parents/guardians at least 2 weeks prior to the school data collection date. Parents were provided contact information if they wished to withdraw their child from the study. All students attending participating schools and not withdrawn from the study by a parent/guardian were considered eligible to participate. Students could choose to not participate or to withdraw at any time. The COMPASS study has received ethical approval from the University of Waterloo Human Research Ethics Committee (ORE#30118), Brock University Research Ethics Committee (REB#18–099), and participating school boards. Additional details regarding study methods can be found online (www.compass.uwaterloo.ca).

### Measures

#### Dietary intake and eating behaviour outcomes

*Daily breakfast* consumption was self-reported with a single item “I eat breakfast every day”. Participants could respond “yes” or leave blank to indicate “no”. The question on breakfast allows participants to select the reason for skipping breakfast (including “Other”); if a reason was indicated, “no” was assumed for daily breakfast consumption and if no reason was indicated, data were treated as missing.

For all other eating behaviours, participants were asked to indicate whether they had engaged in the target behaviour for each day of the past week (Monday through Sunday). Data were treated as missing if all responses for this section were blank.

*Water* consumption was measured by asking participants to indicate days on which they had drunk “water (plain)” during the last week. Due to the low frequencies for responses totaling 1–6 days, data were dichotomized based on whether plain water had been consumed on all 7 days or not.

*Restrictive eating* was measured by asking participants to indicate on which days they had “restrict[ed]/alter[ed] your food intake with the intention of changing your weight/shape?” Based on response distribution, data were dichotomized as to whether the participant had restricted their eating behaviours in the past week or not.

*Purchased snack* consumption was measured by asking participants to indicate on which days they consumed snacks “purchased from a vending machine, corner store, snack bar, or canteen”. Based on response distribution, data were dichotomized as to whether the participant had consumed snacks purchased from these sources in the past week or not.

Frequency of *fast food* consumption was measured by asking participants on which days they had consumed food “purchased at a fast food place or restaurant”. Previous literature has identified two or more occasions of eating fast food per week as “problematic” or “excessive” consumption (Laxer & Janssen, 2014; Pereira et al., 2005); responses were trichotomized as “none”, “one”, or “two or more” occasions, as less than 10% of the sample reported more than 2 days of fast food consumption in the past week.

Frequency of *fruit* consumption was measured by asking participants to indicate on which days they had “eat[en] fruit (fresh, canned, dried or frozen)”.

Frequency of *vegetable* consumption was measured by asking participants to indicate on which days they had “eat[en] vegetables (raw or cooked, fresh, canned, or frozen)”. Fruit and vegetable responses were scored as 0–7 days.

#### Social positions

Student social positions included gender, race/ethnicity, and SES. Gender responses included *male*, *female*, *I describe my gender in a different way* (hereby referred to as gender diverse), and *I prefer not to say*. We have referred to gender but acknowledge the question does not specify whether sex or gender is being asked and male and female may be regarded as sex terms; the measure may elicit gender (Johnson et al., 2009). Race/ethnicity responses allowed individuals to select all that apply of *Asian*, *Black*, *Latin American*, *White*, or *Other*; individuals who identified multiple racial identities were recategorized as “Other”. Consistent with previous research (e.g. Duncan et al., 2023a, b), a SES composite score was derived from four items: (i) whether they sometimes go to bed hungry because there is not enough money to buy food (yes = 0, no = 1); (ii) perceived familial financial comfort relative to students in their class (more comfortable = 2, as comfortable = 1, less comfortable = 0); (iii) whether they have their own bedroom (yes = 1, no = 0); and (iv) whether they were worried about their family paying bills and expenses due to COVID-19 (true/mostly true = 0, neutral or don’t know/false/mostly false = 1). Individuals scoring a 0 were reclassified as a 1 due to low frequency (*n* = 51), resulting in a 5 to 1 scale where 5 represents higher SES and 1 represents lower SES. Having one’s own bedroom is an indicator of material affluence derived from the Family Affluence Scale, which has demonstrated validity in national youth surveys (Boyce et al., 2006). Going to bed hungry because there is not enough money to buy food is an indicator of hunger due to food insecurity (Koyanagi et al., 2019; Smith et al., 2022); comparable single-item measures have demonstrated acceptable sensitivity (85%), specificity (80%), and reliability in comparison to the Household Food Security Scale (Kleinman et al., 2007). Perceived family affluence relative to their classmates’ families provides a salient reference group, and developmentally, adolescents are particularly sensitive to peer comparisons and social standing; adolescents’ perceptions of their family’s economic situation are believed to reflect cumulative experiences (Svedberg et al., 2016). Further, perceptions of being deprived relative to the perceived standard of living are shown to contribute to poor health outcomes in youth beyond material affluence (Elgar et al., 2013). Last, student worry about their family’s ability to pay bills was adapted from the COVID-19 Adolescent Symptom and Psychological Experience Questionnaire (CASPE) (Ladouceur, 2020; De France et al., 2022) to assess pandemic-related impacts on perceived household financial security. Perceived family financial security measures have demonstrated greater validity and reliability in adolescents in comparison to traditional SES indicators of parental/guardian income and education level (Hammond et al., 2021; Svedberg et al., 2016).

#### Confounding variables

Models controlled for education year of the student, province, self-reported school mode (in person, online, or blended [half online, half in-person]), and school area urbanicity (rural, small urban, medium urban, or large urban) and median annual household incomes (≤ $40,000 [approximate national median income level (Statistics Canada, n.d.)], $40,001–$60,000, $60,001–$80,000, and > $80,000), from Statistics Canada’s 2016 census data, based on school postal codes.

### Statistical analysis

Analyses were conducted in SAS v9.4. To assess the relationship between diet/eating variables and social positions, logistic and proportional odds ordered logistic regression models with a random intercept to account for school clustering (i.e. students nested in schools) were generated; diet/eating measures were regressed on social positions and confounding variables. Categorical predictor variables were dummy coded while ordinal variables were orthogonal polynomial coded. Type 3 (or Wald) analyses of effects tests (Wald, 1943) assessed overall group differences for each variable of interest while controlling for all other variables in the model. Pairwise post hoc comparison tests were conducted for statistically significant main effects based on least square means. The Benjamini-Hochberg (Benjamini & Hochberg, 1995) correction for the false discovery rate was used to adjust *P*-values for multiple post hoc comparisons.

## Results

After excluding individuals who did not report their year of education (*n* = 751) or listed education year as “other” (*n* = 580, e.g. Quebec new immigrant classes), the sample available for analysis consisted of 52,138 participants from 133 schools. Table 1 summarizes the social position and confounding variables and Table 2 summarizes all diet/eating outcomes. Mean age of the sample was 15.0 (1.53). Pairwise deletion analysis allowed for 45,175 cases to be modeled for breakfast consumption, and 45,058 cases for all other diet/eating outcomes.

**Table 1:** Sample characteristics of secondary school students who participated in the COMPASS study in the 2020–2021 school year

Variable	*N*	%
Province		
AB	2228	4.3%
BC	5302	10.2%
ON	13,838	26.5%
QC	30,770	59.0%
Education year		
* Secondaire 1*	7591	14.6%
* Secondaire 2*	6621	12.7%
Grade 9/*Secondaire 3*	11,482	22.0%
Grade 10/*Secondaire 4*	11,789	22.6%
Grade 11/*Secondaire 5*	9928	19.0%
Grade 12	4727	9.1%
School area median household income		
≤ $40,000	2593	5.0%
$40,001–$60,000	15,340	29.4%
$60,001–$80,000	25,503	48.9%
> $80,000	8702	16.7%
School mode		
In-person	16,045	30.9%
Online	24,175	46.6%
Blended	11,679	22.5%
Missing	239	-
Gender		
Female	26,726	51.4%
Male	23,299	44.8%
Describe in another way	1205	2.3%
Prefer not to say	788	1.5%
Missing	120	-
Race/ethnicity		
Asian	3860	7.4%
Black	1613	3.1%
Latin American/Hispanic	958	1.8%
Other/multiple	7114	13.6%
White	38,593	74.0%
Urbanicity		
Rural	5988	11.5%
Small urban	15,799	30.3%
Medium urban	4396	8.4%
Large urban	25,955	49.8%
SES score		
1 (lowest)	560	1.2%
2	2239	4.9%
3	7761	16.9%
4	25,356	55.2%
5 (highest)	10,037	21.8%
Missing	6185	-

*N* = 52,138; *SES*, socioeconomic status, based on composite of four items

**Table 2:** Dietary consumption and eating behaviours of secondary school students who participated in the COMPASS study in the 2020–2021 school year

Variable	*N*	%
Daily breakfast		
No	23,464	47.7%
Yes	24,762	50.3%
Missing	3912	-
Water		
0–6 days	6867	14.4%
7 days	40,787	85.6%
Missing	4484	-
Purchased snacks		
0 days	33,664	70.6%
1–7 days	13,990	29.4%
Missing	4484	-
Restricted eating		
0 days	32,804	68.8%
1–7 days	14,850	31.2%
Missing	4484	-
Fast food		
0 days	18,904	39.7%
1 day	16,831	35.3%
2–7 days	11,919	25.0%
Missing	4484	-
Fruits		
0 days	5574	11.7%
1 day	2361	5.0%
2 days	3048	6.4%
3 days	3948	8.3%
4 days	3948	8.3%
5 days	3012	6.3%
6 days	1216	2.6%
7 days	24,547	51.5%
Missing	4484	-
Vegetables		
0 days	4741	9.9%
1 day	1933	4.1%
2 days	2530	5.3%
3 days	3355	7.0%
4 days	3425	7.2%
5 days	2771	5.8%
6 days	1597	3.4%
7 days	27,302	57.3%
Missing	4484	-

Table 3 summarizes analysis of variance tests of overall group differences for all outcomes. Gender was associated with all outcomes except purchased snack consumption. Race/ethnicity and SES score were associated with all dietary intake and eating behaviour outcomes.

**Table 3:** Main effects models for differences in diet/eating outcomes by social positions among secondary school students

Group	Df	Daily breakfast		Water		Restricted eating		Purchased snacks		Fast food		Fruits		Vegetables	
		*χ*^2^	*p*	*χ*^2^	*p*	*χ*^2^	*p*	*χ*^2^	*p*	*χ*^2^	*p*	*χ*^2^	*p*	*χ*^2^	*p*
Gender	2	907.7	** < 0.001**	163.5	** < 0.001**	942.9	** < 0.001**	4.7	0.195	164.6	** < 0.001**	454.7	** < 0.001**	358.2	** < 0.001**
Race/ethnicity	4	115.7	** < 0.001**	98.7	** < 0.001**	57.4	** < 0.001**	107.4	** < 0.001**	55.1	** < 0.001**	110.8	** < 0.001**	307.6	** < 0.001**
SES score	4	370.1	** < 0.001**	111.8	** < 0.001**	298.4	** < 0.001**	55.9	** < 0.001**	5150.6	** < 0.001**	8509.2	** < 0.001**	8229.4	** < 0.001**
*n*		45,175		45,058		45,058		45,058		45,058		45,058		45,058	

Survey data from secondary school students in Alberta, British Columbia, Ontario, and Quebec, Canada, who participated in the COMPASS study in 2020–2021. Reported *n* represents number of complete cases after pairwise deletion. *SES*, socioeconomic status. Results are for type 3 analysis of effects tests of overall group differences for social position, all models adjusted for education year, self-reported school mode, province, and school area urbanicity and median income, gender, race/ethnicity, SES score, and a random intercept for school cluster. *P*-values < 0.05 are bolded

Specific effect size contrasts by gender are provided in Table 4. Females were more likely than males to not eat breakfast daily and to restrict eating. Females were more likely to consume fruit, vegetables, and fast food on more days of the week than males. Gender-diverse students were the least likely to report eating breakfast and drinking plain water daily, and to regularly consume fruit and vegetables; they were also more likely to restrict their eating than males. Individuals who preferred not to report a gender were broadly similar to gender-diverse students in terms of magnitude and direction of effects, albeit effects were slightly less pronounced among those who preferred not to say; the only significant difference between these two groups was for water consumption.

**Table 4:** Gender differences of dietary intake and eating behaviours among secondary school students

Outcomes and contrasts	AOR	95% CI	*z*	*p*_adjusted_
Daily breakfast				
Female: male	0.57	[0.55, 0.60]	− 27.6	** < 0.001**
Female: gender diverse	1.62	[1.34, 1.95]	6.7	** < 0.001**
Female: prefer not to say	1.39	[1.11, 1.75]	3.9	** < 0.001**
Male: gender diverse	2.82	[2.33, 3.41]	14.3	** < 0.001**
Male: prefer not to say	2.43	[1.94, 3.05]	10.3	** < 0.001**
Gender diverse: prefer not to say	0.86	[0.64, 1.15]	− 1.3	0.180
Water				
Female: male	0.99	[0.92, 1.06]	− 0.5	0.640
Female: gender diverse	2.39	[1.96, 2.91]	11.7	** < 0.001**
Female: prefer not to say	1.64	[1.25, 2.14]	4.9	** < 0.001**
Male: gender diverse	2.42	[1.99, 2.95]	11.8	** < 0.001**
Male: prefer not to say	1.66	[1.27, 2.17]	5.0	** < 0.001**
Gender diverse: prefer not to say	0.69	[0.5, 0.95]	− 3.1	**0.002**
Restricted eating				
Female: male	1.96	[1.86, 2.06]	30.6	** < 0.001**
Female: gender diverse	1.11	[0.92, 1.33]	1.5	0.168
Female: prefer not to say	1.20	[0.96, 1.51]	2.1	0.052
Male: gender diverse	0.57	[0.47, 0.68]	− 8.2	** < 0.001**
Male: prefer not to say	0.61	[0.49, 0.77]	− 5.6	** < 0.001**
Gender diverse: prefer not to say	1.09	[0.82, 1.44]	0.8	0.450
*Purchased snack*				
* Female: male*	*0.99*	*[0.94, 1.04]*	* − 0.4*	*0.685*
* Female: gender diverse*	*0.86*	*[0.72, 1.04]*	* − 2.1*	*0.147*
* Female: prefer not to say*	*0.95*	*[0.75, 1.20]*	* − 0.6*	*0.685*
* Male: gender diverse*	*0.87*	*[0.72, 1.05]*	* − 2.0*	*0.147*
* Male: prefer not to say*	*0.96*	*[0.75, 1.21]*	* − 0.5*	*0.685*
* Gender diverse: prefer not to say*	*1.10*	*[0.82, 1.48]*	*0.8*	*0.685*
Fast food				
Female: male	1.09	[1.04, 1.14]	4.7	** < 0.001**
Female: gender diverse	1.05	[0.89, 1.24]	0.8	0.537
Female: prefer not to say	1.17	[0.95, 1.43]	2.0	0.136
Male: gender diverse	0.96	[0.81, 1.14]	− 0.6	0.537
Male: prefer not to say	1.07	[0.87, 1.32]	0.9	0.537
Gender diverse: prefer not to say	1.11	[0.86, 1.44]	1.1	0.536
Vegetables				
Female: male	1.21	[1.15, 1.27]	9.9	** < 0.001**
Female: gender diverse	1.53	[1.30, 1.79]	6.9	** < 0.001**
Female: prefer not to say	1.48	[1.21, 1.81]	5.2	** < 0.001**
Male: gender diverse	1.26	[1.07, 1.48]	3.8	** < 0.001**
Male: prefer not to say	1.23	[1.01, 1.50]	2.7	**0.009**
Gender diverse: prefer not to say	0.97	[0.76, 1.25]	− 0.3	0.769
Fruits				
Female: male	1.31	[1.26, 1.37]	14.7	** < 0.001**
Female: gender diverse	1.6	[1.36, 1.87]	7.8	** < 0.001**
Female: prefer not to say	1.42	[1.16, 1.73]	4.6	** < 0.001**
Male: gender diverse	1.22	[1.04, 1.43]	3.2	**0.002**
Male: prefer not to say	1.08	[0.88, 1.32]	1.0	0.322
Gender diverse: prefer not to say	0.89	[0.69, 1.14]	− 1.3	0.243

Survey data from 52,138 secondary school students in Alberta, British Columbia, Ontario, and Quebec, Canada, who participated in the COMPASS study in 2020–2021. *AOR*, adjusted odds ratio (controlling for education year, school mode, province, regional urbanicity and median income, race/ethnicity, SES score, and a random intercept for school cluster). *CI*, Bonferroni adjusted confidence interval. For dichotomous outcomes, AOR > 1 values indicate higher likelihood of endorsing engaging in daily breakfast or water consumption, restricted eating in the past week, or consuming purchased snacks. For ordered outcomes, AOR > 1 represents higher likelihood of higher frequency of consumption of fast food, fruit, or vegetables per week. *P*-values are adjusted for 3 comparisons with the Benjamini-Hochberg approach, values < 0.05 are bolded. Italicized rows are for outcomes where the Type 3 test was not significant (see Table 2), but contrasts are reported for reference

Effect sizes by race/ethnicity are reported in Table 5. Daily breakfast eating was most likely to be reported by Latin American students, followed by Asian, white, and “Other” students, consecutively, and least likely to be reported by Black students. Black, Latin American, and “Other” students were more likely to restrict their eating, consume purchased snacks, and consume fast food more frequently, in comparison to white and Asian students, and were also less likely to consume plain water daily and eat vegetables on more days of the week. Black students were less likely than any other racial/ethnic group of students to eat vegetables or fruit on more days of the past week. White students were most likely to report a higher frequency of fruit consumption.

**Table 5:** Racial/ethnic differences in dietary intake and eating behaviours among secondary school students

Outcomes and contrasts	AOR	95% CI	*z*	*p*_adjusted_
Daily breakfast				
Asian: Black	1.64	[1.34, 2.02]	6.7	** < 0.001**
Asian: Latin	0.80	[0.63, 1.02]	− 2.6	**0.012**
Asian: Other	1.33	[1.15, 1.53]	5.7	** < 0.001**
Asian: White	1.08	[0.95, 1.23]	1.6	0.100
Black: Latin	0.49	[0.37, 0.64]	− 7.5	** < 0.001**
Black: Other	0.81	[0.67, 0.97]	− 3.2	**0.002**
Black: White	0.66	[0.55, 0.78]	− 6.8	** < 0.001**
Latin: Other	1.65	[1.32, 2.07]	6.3	** < 0.001**
Latin: White	1.34	[1.08, 1.66]	3.8	** < 0.001**
Other: White	0.81	[0.74, 0.89]	− 6.8	** < 0.001**
Water				
Asian: Black	1.96	[1.48, 2.58]	6.8	** < 0.001**
Asian: Latin	1.76	[1.28, 2.43]	4.9	** < 0.001**
Asian: Other	1.50	[1.22, 1.84]	5.5	** < 0.001**
Asian: White	1.18	[0.97, 1.43]	2.4	**0.023**
Black: Latin	0.90	[0.64, 1.26]	− 0.9	0.378
Black: Other	0.77	[0.61, 0.96]	− 3.3	**0.002**
Black: White	0.60	[0.49, 0.74]	− 6.7	** < 0.001**
Latin: Other	0.85	[0.64, 1.13]	− 1.6	0.125
Latin: White	0.67	[0.51, 0.88]	− 4.1	** < 0.001**
Other: White	0.79	[0.70, 0.88]	− 5.9	** < 0.001**
Restricted eating				
Asian: Black	0.71	[0.58, 0.88]	− 4.5	** < 0.001**
Asian: Latin	0.78	[0.61, 0.99]	− 2.8	**0.010**
Asian: Other	0.81	[0.70, 0.94]	− 4.0	** < 0.001**
Asian: White	0.96	[0.84, 1.10]	− 0.8	0.499
Black: Latin	1.10	[0.83, 1.44]	0.9	0.439
Black: Other	1.14	[0.94, 1.37]	1.9	0.081
Black: White	1.35	[1.13, 1.62]	4.8	** < 0.001**
Latin: Other	1.04	[0.83, 1.31]	0.5	0.645
Latin: White	1.24	[0.99, 1.54]	2.7	**0.011**
Other: White	1.19	[1.09, 1.30]	5.5	** < 0.001**
Purchased snacks				
Asian: Black	0.64	[0.51, 0.79]	− 5.8	** < 0.001**
Asian: Latin	0.63	[0.49, 0.81]	− 5.2	** < 0.001**
Asian: Other	0.77	[0.67, 0.90]	− 4.8	** < 0.001**
Asian: White	0.96	[0.84, 1.11]	− 0.8	0.489
Black: Latin	0.99	[0.75, 1.31]	− 0.1	0.938
Black: Other	1.22	[1.01, 1.48]	2.9	**0.006**
Black: White	1.51	[1.27, 1.81]	6.5	** < 0.001**
Latin: Other	1.23	[0.97, 1.54]	2.5	**0.016**
Latin: White	1.53	[1.22, 1.90]	5.4	** < 0.001**
Other: White	1.24	[1.14, 1.36]	6.7	** < 0.001**
Fast food				
Asian: Black	0.81	[0.67, 0.97]	− 3.2	**0.002**
Asian: Latin	0.72	[0.58, 0.90]	− 4.2	** < 0.001**
Asian: Other	0.85	[0.75, 0.97]	− 3.5	**0.001**
Asian: White	0.99	[0.88, 1.11]	− 0.3	0.728
Black: Latin	0.90	[0.70, 1.14]	− 1.3	0.263
Black: Other	1.06	[0.89, 1.26]	0.9	0.387
Black: White	1.22	[1.04, 1.43]	3.6	**0.001**
Latin: Other	1.18	[0.96, 1.44]	2.3	**0.030**
Latin: White	1.36	[1.13, 1.65]	4.5	** < 0.001**
Other: White	1.16	[1.07, 1.25]	5.2	** < 0.001**
Fruits				
Asian: Black	1.50	[1.25, 1.81]	6.2	** < 0.001**
Asian: Latin	1.01	[0.82, 1.26]	0.2	0.934
Asian: Other	1.01	[0.89, 1.14]	0.2	0.934
Asian: White	0.88	[0.78, 0.99]	− 3.0	**0.004**
Black: Latin	0.67	[0.53, 0.86]	− 4.6	** < 0.001**
Black: Other	0.67	[0.57, 0.79]	− 6.8	** < 0.001**
Black: White	0.59	[0.50, 0.68]	− 9.7	** < 0.001**
Latin: Other	0.99	[0.81, 1.22]	− 0.1	0.934
Latin: White	0.87	[0.72, 1.05]	− 2.0	0.058
Other: White	0.87	[0.81, 0.95]	− 4.8	** < 0.001**
Vegetables				
Asian: Black	2.22	[1.84, 2.69]	11.8	** < 0.001**
Asian: Latin	1.53	[1.23, 1.90]	5.5	** < 0.001**
Asian: Other	1.17	[1.02, 1.33]	3.3	**0.001**
Asian: White	0.93	[0.83, 1.06]	− 1.5	0.126
Black: Latin	0.69	[0.54, 0.87]	− 4.4	** < 0.001**
Black: Other	0.52	[0.44, 0.62]	− 10.8	** < 0.001**
Black: White	0.42	[0.36, 0.49]	− 15.6	** < 0.001**
Latin: Other	0.76	[0.62, 0.93]	− 3.8	** < 0.001**
Latin: White	0.61	[0.50, 0.74]	− 7.2	** < 0.001**
Other: White	0.80	[0.74, 0.87]	− 7.8	** < 0.001**

Survey data from 52,138 secondary school students in Alberta, British Columbia, Ontario, and Quebec, Canada, who participated in the COMPASS study in 2020–2021. *AOR*, adjusted odds ratio (controlling for education year, school mode, province, school area urbanicity and median income, gender, SES score, and a random intercept for school cluster). *CI*, Bonferroni adjusted confidence interval. For dichotomous outcomes, AOR > 1 values indicate higher likelihood of endorsing engaging in daily breakfast or water consumption, restricted eating in the past week, or consuming snacks. For ordered outcomes, AOR > 1 represents higher likelihood of higher frequency of consumption of fast food, fruit, or vegetables per week. *P*-values are adjusted for 10 comparisons with the Benjamini-Hochberg approach, values < 0.05 are bolded

Results by specific SES score are presented in Table 6. Lower SES scores were associated with lower odds of consuming breakfast and water daily and fewer days of fruit and vegetable consumption. Lower SES was also associated with higher odds of restricted eating and purchased snack consumption; differences in snack consumption tended to plateau at either end of the scale where scores of 1–3 resulted in similar odds, while scores of 4 or 5 were similar. Results were less consistent for fast food consumption by SES scores, which followed a somewhat U-shaped pattern: individuals in the middle of the SES scale (around a score of 3 or 4) were least likely to consume fast food more frequently, whereas high frequency of fast food consumption was most likely among the lowest and highest scores (1 and 5, respectively).

**Table 6:** Socioeconomic score differences in dietary intake and eating behaviours among secondary school students

Outcomes and contrasts	AOR	95% CI	*z*	*p*_adjusted_
Daily breakfast				
1: 2	0.74	[0.54, 1.01]	− 2.8	**0.006**
1: 3	0.49	[0.37, 0.66]	− 6.9	** < 0.001**
1: 4	0.39	[0.29, 0.51]	− 9.4	** < 0.001**
1: 5	0.35	[0.26, 0.46]	− 10.3	** < 0.001**
2: 3	0.67	[0.58, 0.77]	− 7.8	** < 0.001**
2: 4	0.52	[0.45, 0.60]	− 13.4	** < 0.001**
2: 5	0.47	[0.41, 0.54]	− 14.8	** < 0.001**
3: 4	0.78	[0.72, 0.84]	− 9.0	** < 0.001**
3: 5	0.71	[0.64, 0.77]	− 10.9	** < 0.001**
4: 5	0.90	[0.84, 0.97]	− 4.2	** < 0.001**
Water				
1: 2	0.73	[0.52, 1.01]	− 2.7	**0.007**
1: 3	0.64	[0.47, 0.87]	− 4.1	** < 0.001**
1: 4	0.52	[0.38, 0.69]	− 6.3	** < 0.001**
1: 5	0.47	[0.34, 0.63]	− 7.1	** < 0.001**
2: 3	0.88	[0.74, 1.06]	− 1.9	0.056
2: 4	0.71	[0.60, 0.84]	− 5.7	** < 0.001**
2: 5	0.64	[0.53, 0.77]	− 6.8	** < 0.001**
3: 4	0.80	[0.72, 0.89]	− 5.9	** < 0.001**
3: 5	0.73	[0.64, 0.82]	− 7.1	** < 0.001**
4: 5	0.90	[0.81, 1.01]	− 2.8	**0.007**
Restricted eating				
1: 2	0.94	[0.71, 1.24]	− 0.7	0.505
1: 3	1.14	[0.88, 1.48]	1.4	0.175
1: 4	1.67	[1.30, 2.16]	5.7	** < 0.001**
1: 5	1.46	[1.12, 1.89]	4.1	** < 0.001**
2: 3	1.22	[1.06, 1.40]	3.9	** < 0.001**
2: 4	1.79	[1.57, 2.04]	12.4	** < 0.001**
2: 5	1.56	[1.35, 1.79]	8.9	** < 0.001**
3: 4	1.47	[1.36, 1.59]	13.5	** < 0.001**
3: 5	1.28	[1.17, 1.40]	7.4	** < 0.001**
4: 5	0.87	[0.81, 0.94]	− 5.2	** < 0.001**
Purchased snacks				
1: 2	1.13	[0.85, 1.50]	1.2	0.270
1: 3	1.16	[0.89, 1.51]	1.5	0.178
1: 4	1.39	[1.07, 1.80]	3.5	**0.001**
1: 5	1.34	[1.02, 1.74]	3.1	**0.004**
2: 3	1.03	[0.89, 1.19]	0.5	0.613
2: 4	1.23	[1.07, 1.41]	4.2	**0.000**
2: 5	1.19	[1.02, 1.37]	3.3	**0.002**
3: 4	1.20	[1.10, 1.30]	6.1	** < 0.001**
3: 5	1.15	[1.05, 1.27]	4.2	** < 0.001**
4: 5	0.96	[0.89, 1.04]	− 1.3	0.227
Fast food				
1: 2	1.04	[0.80, 1.35]	0.4	0.779
1: 3	1.14	[0.90, 1.46]	1.6	0.170
1: 4	1.19	[0.94, 1.51]	2.1	0.081
1: 5	0.99	[0.77, 1.26]	− 0.2	0.877
2: 3	1.10	[0.97, 1.26]	2.2	0.078
2: 4	1.15	[1.02, 1.30]	3.2	**0.004**
2: 5	0.95	[0.84, 1.08]	− 1.1	0.349
3: 4	1.04	[0.97, 1.12]	1.6	0.170
3: 5	0.86	[0.79, 0.94]	− 5.1	** < 0.001**
4: 5	0.83	[0.78, 0.88]	− 8.4	** < 0.001**
Fruits				
1: 2	0.75	[0.59, 0.96]	− 3.2	**0.001**
1: 3	0.59	[0.47, 0.74]	− 6.5	** < 0.001**
1: 4	0.50	[0.40, 0.62]	− 8.8	** < 0.001**
1: 5	0.37	[0.29, 0.46]	− 12.3	** < 0.001**
2: 3	0.79	[0.69, 0.89]	− 5.5	** < 0.001**
2: 4	0.66	[0.58, 0.74]	− 10.3	** < 0.001**
2: 5	0.49	[0.43, 0.55]	− 16.3	** < 0.001**
3: 4	0.84	[0.78, 0.90]	− 7.3	** < 0.001**
3: 5	0.62	[0.57, 0.68]	− 16.0	** < 0.001**
4: 5	0.75	[0.70, 0.80]	− 12.5	** < 0.001**
Vegetables				
1: 2	0.66	[0.52, 0.85]	− 4.7	** < 0.001**
1: 3	0.57	[0.46, 0.72]	− 6.8	** < 0.001**
1: 4	0.46	[0.37, 0.58]	− 9.6	** < 0.001**
1: 5	0.38	[0.30, 0.47]	− 12.0	** < 0.001**
2: 3	0.87	[0.76, 0.98]	− 3.2	**0.002**
2: 4	0.70	[0.62, 0.79]	− 8.4	** < 0.001**
2: 5	0.57	[0.50, 0.64]	− 12.5	** < 0.001**
3: 4	0.81	[0.75, 0.87]	− 8.3	** < 0.001**
3: 5	0.65	[0.60, 0.71]	− 13.9	** < 0.001**
4: 5	0.81	[0.76, 0.87]	− 8.7	** < 0.001**

Survey data were from 52,138 secondary school students in Alberta, British Columbia, Ontario, and Quebec, Canada, who participated in the COMPASS study in 2020–2021. Socioeconomic status scores range from 1 (lowest) to 5 (highest) based on composite of four items. *AOR*, adjusted odds ratio (controlling for education year, school mode, province, school area urbanicity and median income, gender, race/ethnicity, and a random intercept for school cluster). *CI*, Bonferroni adjusted confidence interval. For dichotomous outcomes, AOR > 1 values indicate higher likelihood of endorsing engaging in daily breakfast or water consumption, restricted eating in the past week, or consuming purchased snacks. For ordered outcomes, AOR > 1 represents higher likelihood of being higher frequency of consumption of fast food, fruit, or vegetables per week. *P*-values are adjusted for 3 comparisons with the Benjamini-Hochberg approach, values < 0.05 are bolded

## Discussion

Consistent disparities in dietary intake and eating behaviours were found by gender, race/ethnicity, and socioeconomic status in a large sample of secondary school students attending secondary schools in the four largest Canadian provinces (Alberta, British Columbia, Ontario, and Quebec) in 2020–2021, the first full academic year post-COVID-19 onset. Gender diverse, those who preferred not to indicate gender, Black and Latin American, and lower SES students were generally the least likely to report responses in line with current dietary recommendations across the majority of included measures relative to their male, white and Asian, and higher SES peers, respectively.

Females reported more favourable fruit and vegetable intakes than males. Likewise, females exceeded males in fruit and/or vegetable intake in both the 2017/2018 Health Behaviour of School-aged Children (HBSC) study (WHO, 2020) and the 2014 and 2021 cycles of the Canadian Community Health Survey (CCHS) (Rao et al., 2017; Statistics Canada, 2022). However, females had less healthful responses for restrictive eating, breakfast skipping, and fast food consumption relative to males, and no difference in daily plain water or purchased snack intake. Our results for fast food consumption may reflect the greater tendency to use food and lunch times for fitting in and socializing with peers among females relative to their male counterparts (Deslippe et al., 2021). Inconsistent findings exist on gender differences in adolescent water consumption. In the 2015 CCHS, 89% of males aged 14–18 reported previous-day water consumption, compared to 87% of females, up from 77% and 74% in 2004, respectively (Garriguet, 2019). In contrast, a study of New Brunswick students found girls consumed more water than boys (Doggui et al., 2022), and in a US study, males were more likely than females to drink at least three glasses of plain water per day (Michael et al., 2023).

Our gender and restrictive eating and breakfast skipping results align with previous research (Bishop et al., 2020; Patte & Leatherdale, 2017; Lazzeri et al., 2016; Michael et al., 2023; Rao et al., 2017; WHO, 2020), and were as expected based on sociocultural body ideals and gender norms, including feminine ideals of thinness and masculine ideals of muscularity (Deslippe et al., 2021). Adolescents have heightened exposure and vulnerability to such influences, which were potentially amplified during the pandemic; screen media is rife with diet culture and weight-normative messages (e.g. #quarantine15), alongside food marketing (Harris et al., 2021; Lucibello et al., 2021). Given gendered variations in weight/shape goals, our restrictive eating measure did not focus exclusively on dieting for weight loss but was framed in an attempt to capture any alteration of diet and eating with the intention to change weight or shape; however, female, gender diverse, and prefer not to say participants still surpassed male students on this outcome.

Gender-diverse students and those who preferred not to report gender were the least likely to report daily water and regular fruit and vegetable intake, and more likely to report restrictive eating than males. Scarce evidence exists on dietary intake and eating behaviours among transgender and gender-diverse adolescents. Our results align with a 2016 survey of US students in Grades 9/10, in which transgender and gender-nonconforming students reported more meal skipping, greater fast food and soft drink intake, and less frequent consumption of fruit and water than their cisgender peers (Bishop et al., 2020). There is a clear need for further research on dietary intake and eating behaviours in transgender and gender-diverse populations.

Disparities by race/ethnicity and SES scores were generally consistent across outcomes. Overall, Black students reported the least favourable responses to all dietary measures of students, generally followed by Latin American students, while white and Asian students were most likely to report more healthful dietary intake and behaviours. The one exception was for breakfast consumption, with Latin American students the most likely to eat breakfast daily. Results generally align with evidence of racial and ethnic disparities in US secondary school students; in a 2021 nationally representative survey, poor dietary behaviours were highest among Black students and lowest among Asian students, relative to students from other groups (Michael et al., 2023). US research points to disproportionate exposure to food insecurity (Morales et al., 2021; Odoms-Young & Bruce, 2018) and targeted food marketing (Harris et al., 2021) among Black populations and persons of colour.

Lower SES scores were consistently associated with lower odds of recommended dietary intake and behaviours across outcomes. Results resemble pre-pandemic data from Canadian adolescents. In the 2014 CCHS and 2012/13 Canadian Health Measures Survey, adolescents from higher-income adequacy households had a higher prevalence of consuming five or more fruits and vegetables a day than their peers from low- or moderate-income adequacy households (Rao et al., 2017). Similarly, in the 2017/2018 HBSC study, higher family affluence was associated with higher fruit, vegetable, and daily breakfast consumption (WHO, 2020). One of the strongest correlates of fruit and vegetable intake in adolescents is home availability, which is associated with food security and SES (Neumark-Sztainer et al., 2003). Increased food insecurity, higher food prices, and the interruption of school food programs may have exacerbated socioeconomic inequities during the COVID-19 pandemic. Based on the Canadian Income Survey, 18% of families experienced some level of food insecurity over the past year in 2022, up from 16% in 2021 and 17% in 2020, with varied rates across Canada (Statistics Canada, 2023). A systematic review found that children living in low-income households were experiencing the negative nutritional ramifications of the lockdowns at higher levels due to lack of access to school programs they relied on for daily meals prior to the pandemic (Chaabane et al., 2021).

The one exception found for SES-dietary patterns was the consumption of fast food. A u-shaped association indicated that students with SES scores in the middle were least likely to consume fast food, while their peers at both low and high SES scores were more likely. Lower-income employment tends to have less flexible and regular work schedules (Gerstel & Clawson, 2018), which impedes home-cooked meals (Devine et al., 2009; Dwyer et al., 2015; Jones, 2018). Food swamps and deserts in lower SES neighbourhoods may also play a role, defined as a high density of unhealthy options (e.g. fast food) and scarce access to healthier alternatives (e.g. fresh produce), respectively (Balcaen & Storie, 2018; Robitaille & Paquette, 2020). The higher odds of consuming fast food are relatively less concerning among higher SES adolescents, as their responses to the other studied outcomes were more likely to align with dietary recommendations, suggesting more healthful overall dietary patterns.

Improved nutrition and reduced inequalities are among the United Nations Sustainable Development Goals, 17 interconnected targets to achieve by 2030 for a better and more sustainable future for all. Likewise, the United Nations Convention on the Rights of the Child (CRC) recognizes that children have a right to healthy and affordable food and adequate nutrition (United Nations, 2019). Canada ratified the CRC in 1991, holding duty bearers responsible for protecting these rights. However, many Canadian children experience food insecurity (Statistics Canada, 2023), and Canada remains the only high-income country without a national school food program. That said, while school food programs may help ensure students receive a healthy meal on weekdays, they will not mitigate the risk of food insecurity. Food insecurity is an income issue, and requires policy responses (Men et al., 2021). Despite acknowledging that dietary choices are constrained by contextual factors, responses have historically been limited to stopgap measures (e.g. food banks) or interventions that place the responsibility on individuals (e.g. educational campaigns), rather than addressing the structures and systems that drive the social patterning of health behaviours and outcomes (Alvaro et al., 2011; Salas et al., 2017).

### Limitations

COMPASS was not designed to be provincially or nationally representative. While passive consent protocols (Spence et al., 2014), full school samples, and the inclusion of diverse schools (e.g. private and public schools in areas with varied median household incomes and urbanicity) help support generalizability, the results may not be reflective of all Canadian students. Students and schools were from four provinces. Also, while this study controlled for province and urbanicity, further research should examine whether findings differ by region, given varied social policies and access to resources. Self-report dietary measures are susceptible to recall error and social desirability bias. We were limited to the measures available from the 2020–2021 student questionnaire, which was designed to be brief to improve data quality, but may not capture all important facets of adolescent diets (e.g. sugar-sweetened beverages) or relevant confounders. The water item refers to “plain” water, but Canada’s Food Guide does include fruit and herb-infused water or carbonated water as ways to make water the drink of choice (Health Canada, 2019). Psychometric analysis has not been conducted for the cumulative SES score. The score has been used in previous research (Duncan et al., 2023a, b), and the individual items comprising the SES score were derived and/or adapted from measures considered developmentally appropriate and demonstrating validity and reliability in adolescent populations (e.g. Boyce et al., 2006; Kleinman et al., 2007; Koyanagi et al., 2019; Ladouceur, 2020; Smith et al., 2022), for which traditional SES indicators (e.g. household income, parental/guardian education level) fare poorly in psychometric analyses (Hammond et al., 2021; Svedberg et al., 2016). Given the many comparisons presented in the results, we did not examine differences by individual SES score items or intersections of social positions. Thus, we are unable to determine how results generalize when considering that individuals belong to multiple interconnected social positions. Last, the gender measure may conflate sex and gender. Future research using the revised sex and gender measures introduced in COMPASS will allow analyses across and within cisgender and transgender and gender-diverse populations.

## Conclusion

Adolescents belonging to social positions associated with less power and privilege were less likely to report diets and eating behaviours in line with recommendations, thus placing them at disproportionate risk of future chronic diseases. Results support the critical need to address the socioeconomic and political factors that drive health inequities and to prevent short- and long-term impacts of less favourable diets on the health and development of adolescents from equity-denied groups.

## Contributions to knowledge

What does this study add to existing knowledge?

- Results provide contemporary evidence of inequities in dietary intake and eating behaviour during the first full academic year post-COVID-19 onset in a large sample of adolescents in four Canadian provinces.

What are the key implications for public health interventions, practice, or policy?

- There is a critical need to address the social, economic, and political factors that drive dietary and eating behaviour inequities by gender, race/ethnicity, and SES in adolescents.
- Public health interventions are needed to prevent the short- and long-term impacts of less favourable diets on the health and development of adolescents from equity-denied groups.

## Data Availability

COMPASS study data are available upon request through completion and approval of an online form: https://uwaterloo.ca/compass-system/information-researchers/data-usage-application. The datasets used during the current study are available from the corresponding author on reasonable request.
